# Meiotic Recombination Intermediates Are Resolved with Minimal Crossover Formation during Return-to-Growth, an Analogue of the Mitotic Cell Cycle

**DOI:** 10.1371/journal.pgen.1002083

**Published:** 2011-05-26

**Authors:** Yaron Dayani, Giora Simchen, Michael Lichten

**Affiliations:** 1 Laboratory of Biochemistry and Molecular Biology, Center for Cancer Research, National Cancer Institute, Bethesda, Maryland, United States of America; 2 Department of Genetics, Hebrew University of Jerusalem, Jerusalem, Israel; The University of North Carolina at Chapel Hill, United States of America

## Abstract

Accurate segregation of homologous chromosomes of different parental origin (homologs) during the first division of meiosis (meiosis I) requires inter-homolog crossovers (COs). These are produced at the end of meiosis I prophase, when recombination intermediates that contain Holliday junctions (joint molecules, JMs) are resolved, predominantly as COs. JM resolution during the mitotic cell cycle is less well understood, mainly due to low levels of inter-homolog JMs. To compare JM resolution during meiosis and the mitotic cell cycle, we used a unique feature of *Saccharomyces cerevisiae*, return to growth (RTG), where cells undergoing meiosis can be returned to the mitotic cell cycle by a nutritional shift. By performing RTG with *ndt80* mutants, which arrest in meiosis I prophase with high levels of interhomolog JMs, we could readily monitor JM resolution during the first cell division of RTG genetically and, for the first time, at the molecular level. In contrast to meiosis, where most JMs resolve as COs, most JMs were resolved during the first 1.5–2 hr after RTG without producing COs. Subsequent resolution of the remaining JMs produced COs, and this CO production required the Mus81/Mms4 structure-selective endonuclease. RTG in *sgs1-ΔC795* mutants, which lack the helicase and Holliday junction-binding domains of this BLM homolog, led to a substantial delay in JM resolution; and subsequent JM resolution produced both COs and NCOs. Based on these findings, we suggest that most JMs are resolved during the mitotic cell cycle by dissolution, an Sgs1 helicase-dependent process that produces only NCOs. JMs that escape dissolution are mostly resolved by Mus81/Mms4-dependent cleavage that produces both COs and NCOs in a relatively unbiased manner. Thus, in contrast to meiosis, where JM resolution is heavily biased towards COs, JM resolution during RTG minimizes CO formation, thus maintaining genome integrity and minimizing loss of heterozygosity.

## Author Summary

Cell proliferation involves DNA replication followed by a mitotic division, producing two cells with identical genomes. Diploid organisms, which contain two genome copies per cell, also undergo meiosis, where DNA replication followed by two divisions produces haploid gametes, the equivalent sperm and eggs, with a single copy of the genome. During meiosis, the two copies of each chromosome are brought together and connected by recombination intermediates (joint molecules, JMs) at sites of sequence identity. During meiosis, JMs frequently resolve as crossovers, which exchange flanking sequences, and crossovers are required for accurate chromosome segregation. JMs also form during the mitotic cell cycle, but resolve infrequently as crossovers. To understand how JMs resolve during the mitotic cell cycle, we used a property of budding yeast, return to growth (RTG), in which cells exit meiosis and resume the mitotic cell cycle. By returning to growth cells with high levels of JMs, we determined how JMs resolve in a mitotic cell cycle-like environment. We found that, during RTG, most JMs are taken apart without producing crossovers by Sgs1, a DNA unwinding enzyme. Because Sgs1 is homologous to the mammalian BLM helicase, it is likely that similar mechanisms reduce crossover production in mammals.

## Introduction

Recombination has a major role during meiosis, as it is necessary for accurate homolog segregation at the first meiotic division [1]. Meiotic recombination is initiated by DNA double strand breaks (DSBs) that are formed by the Spo11 nuclease [2], [3]. Single stranded DNA, produced at break ends by 5′ to 3′ resection [4], then interacts with complementary sequences on the homolog or on the sister chromatid [5], [6]. Some interhomolog recombination events produce a noncrossover (NCO), in which both interacting chromosomes retain parental flanking sequence configurations, whereas other events produce a reciprocal exchange of flanking sequences, or crossover (CO). COs, in combination with sister chromatid cohesion, form the inter-homolog linkage that is required for proper homolog segregation [1]. In *Saccharomyces cerevisiae*, COs comprise about one half of all interhomolog recombination events [7]. Meiotic COs are produced by the resolution of joint molecule (JM) intermediates [8]–[10], most of which contain two Holliday junctions [11], here called double Holliday junction JMs (dHJ-JMs).

In most organisms, including *S. cerevisiae*, meiotic DSB formation and recombination are also necessary for progressive colocalization and alignment of homologs during prophase. This process culminates at pachytene, where homologs are joined at sites of recombination and linked tightly along their entire length by a meiosis-specific tripartite protein structure called the synaptonemal complex (SC; [12]).

Although genome-wide programmed DSB formation is central to normal meiosis, it does not usually occur during the mitotic cell cycle. During the budding yeast mitotic cell cycle, most breaks are repaired by recombination between sister chromatids [13]–[15], and the inter-homolog homologous recombination (HR) events that do occur during the mitotic cell cycle produce COs less frequently than in meiosis [13], [16].

The lower yield of COs during mitotic recombination, as compared to meiotic recombination, can be explained in two ways. First, fewer dHJ-JMs are produced per DSB repair event during mitosis than during meiosis [15], and it is possible that most mitotic DSB repair does not involve dHJ-JM formation. Second, it is possible that JMs are produced at significant levels during mitotic HR, but are resolved differently than are JMs produced during meiosis. In *S. cerevisiae*, most meiotic JMs are resolved as COs [8]–[10] in a process that most likely involves endonuclease cleavage of Holliday junctions, and that is triggered by Cdc5, the budding yeast polo-like kinase homolog [17], [10]. Much less is known about JM resolution during the mitotic cell cycle, since the products of intersister recombination cannot be distinguished from the precursor molecules.

Several structure-selective nucleases have been suggested as having a role in JM resolution by Holliday junction cleavage [18]. The most extensively studied of these is a structure-selective heterodimeric endonuclease, hereafter called the Mus81 complex, that contains the conserved Mus81 nuclease in complex with a second protein, called Mms4 in *S*. *cerevisiae* and *Drosophila*, and Eme1 in fission yeast, mammals and plants [19]–[21]. Meiotic progression defects are evident in *S. pombe* and *S. cerevisiae* mutants lacking the Mus81 complex, but the nature of these defects differs in the two organisms. In *S. pombe*, mutants lacking the Mus81 complex show a strong CO defect and accumulate unresolved JMs [19],[22]–[24], while in *S. cerevisiae*, *mus81* or *mms4* mutants show only a minor CO loss and resolve the vast majority of JMs [25]–[29]. Thus, in budding yeast, most meiotic JMs must be resolved by other, yet unidentified endonucleases. It also is not clear whether or not the Mus81 complex resolves JMs that form during the mitotic cell cycle. A recent study of I-Sce1 endonuclease-promoted mitotic recombination in *S. cerevisiae* suggested redundant roles for the Mus81 complex and for the Yen1 endonuclease in interhomolog CO formation [30], but it remains to be established that these crossovers are produced by dHJ-JM resolution.

dHJ-JMs can also be resolved by an endonuclease-independent process, called dissolution, that uses a RecQ-family helicase and a type 1 topoisomerase to disassemble JMs and to produce only NCOs [31]–[34]. Dissolution has been demonstrated in biochemical studies of the human BLM helicase combined with the TOPOIIIα/BLAP75 heterodimer, and of the corresponding budding yeast proteins Sgs1 and Top3/Rmi1 [35], [33], [36]. Dissolution has not yet been directly demonstrated *in vivo*, but is consistent with observations that loss of BLM or Sgs1 helicase activity is accompanied by a substantial increase in mitotic sister chromatid exchange [37]–[39], and that *sgs1* mutants show increased JM accumulation and CO formation during mitotic DSB repair [16], [15]. During meiosis, *sgs1* single mutants show only a slight increase in COs, but produce “abnormal” JMs involving 3 or 4 chromatids at elevated levels [40], [41]. In addition, the CO and JM formation defects of mutants lacking SC components are partially suppressed by *sgs1* mutation [40], [42], [41]. These findings are consistent with the suggestion that the Sgs1/BLM helicase prevents COs by reducing JM levels. However, because this helicase also has the potential to disassemble early strand invasion intermediates that are precursors to JMs [43], [44], it remains to be determined if Sgs1/BLM act primarily to prevent JM formation, or to disassemble JMs once they form.

Finally, JMs that form during the G1 phase of the mitotic cell cycle can, in theory, also be resolved passively by chromosome replication [45], producing a CO if the original JM contains an odd number of HJs and an NCO if the original JM contains an even number of HJs.

In the current study, we present experiments aimed at examining how JMs are resolved during the *S. cerevisiae* mitotic cell cycle. Although several groups have detected JMs in *S. cerevisiae* undergoing vegetative growth [46], [47], [15], definitive study of their resolution has been precluded by their relatively low levels and by the fact that most form between sister chromatids. However, interhomolog JMs can be recovered at high levels during meiosis, especially in cells that lack Ndt80, a transcription factor required for expression of many mid- and late-meiosis proteins, including the Cdc5 polo-like kinase which is required for meiotic JM resolution [48], [17]. *ndt80* mutant cells arrest at the pachytene stage of meiosis, with duplicated but unseparated spindle pole bodies [49], with homologs tightly paired by SC [49], and, most important to this study, with a high level of unresolved JMs [8]. To examine resolution of these JMs in a cellular environment that mimics the mitotic cell cycle, we used a singular property of *S. cerevisiae*, called return to growth (RTG). When cells in meiosis I prophase are shifted to rich medium, they rapidly exit meiosis, adopt a G1-like transcription pattern, and ultimately resume the mitotic cell cycle [50]–[58].

We report here the first molecular characterization of JM resolution during RTG. We show here that, unlike in meiosis, most JMs are resolved after RTG in a manner that does not produce COs. Examination of JM resolution in *sgs1* and in *mus81* mutants suggest that, during RTG of wild-type cells, the majority of JMs are resolved by Sgs1-mediated dissolution, with a minor fraction of JMs being resolved by Mus81 complex-dependent cleavage to produce both CO and NCO products.

## Results

**Figure 1:**
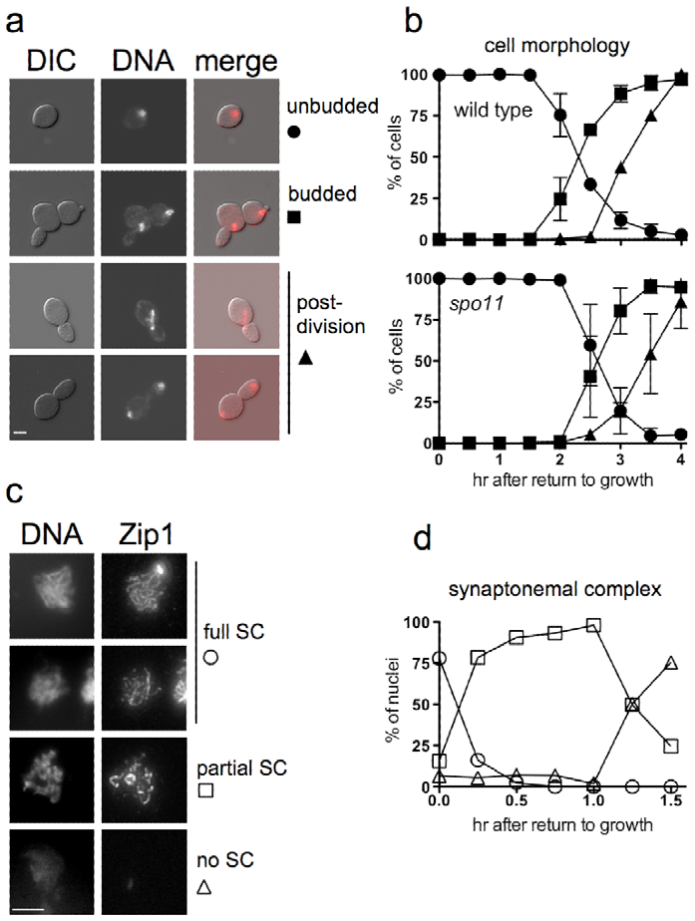
Cell cycle progression and SC breakdown after RTG. a. Representative images of *ndt80* cells (MJL3430) at various stages of RTG, visualized by differential interference contrast (DIC) or by DAPI-staining to detect nuclei (DNA). Note that the daughter cell is elongated as compared to the round mother cell. Scale bar—4µm. b. Time of bud emergence and nuclear division after RTG using *SPO11 ndt80*Δ (MJL3164, top) or *spo11-Y135F ndt80Δ* (MJL2807, bottom); the latter do not form SC or JMs. Circles – unbudded cells; squares – cells with a bud and one nucleus; triangles – cells that are undergoing or have finished nuclear division. Values for MJL3164 are from 4 independent determinations. c. SC breakdown upon RTG. Nuclei (MJL3163) were surface-spread and probed with anti-Zip1 antisera. Representative images of nuclei classified as full SC (long, continuous Zip1 lines), partial SC (discontinuous or dotty Zip1) and no SC (no Zip1 chromosomal staining) are shown together with DNA staining. Extrachromosomal Zip1 aggregates (polycomplex) were also detected as a bright-staining body. Scale bar—4 µm. d. Time of SC breakdown after RTG (MJL3163). At least 150 nuclei were scored for each time point. Circles – nuclei with full SC; squares – nuclei with partial SC; triangles – nuclei with no SC. Values are from a single experiment.

To determine how JMs are resolved after RTG, we used *ndt80Δ* mutant cells, which arrest at pachytene with fully-formed SC and high levels of JMs [49], [8]. In general, RTG experiments involved incubating *ndt80Δ* cells in nutrient-poor sporulation medium (1% potassium acetate) for 7 hr to allow cells to initiate meiosis and arrest at pachytene, and then shifting cells to nutrient-rich growth medium (YPD) to induce RTG. We confirmed that *ndt80*Δ cells retain viability after RTG [49]; virtually all cells produced colonies when a culture incubated 7 hours in sporulation medium was plated on YPD agar plates (colonies/visible cells = 1.0+/−0.1; strain MJL3164—see Table S1). To examine the timing and efficiency of RTG in greater detail, we monitored progression of the first cell cycle after RTG (Figure 1). Budded cells were first observed 2 hr after RTG, and half of the cells had produced a bud by 2.5 hr. Nuclear division occurred about 1 hr after bud emergence, with half of the cells having undergone nuclear division by 3.5 hr after RTG. By 4 hr after RTG, virtually all cells had undergone nuclear division, consistent with the high viability seen in plating experiments.

Cells of the SK1 strain background used here complete a mitotic cell cycle every 80 minutes while growing in YPD (M. L., unpublished data), whereas in the current experiments, the first cell division did not occur until at least 2.5 hr after the shift from sporulation to YPD growth medium (Figure 1b). This difference might be explained if nuclear division during RTG was delayed by the presence of unresolved interhomolog connections that were formed during meiosis. To test this suggestion, we examined RTG in *spo11* mutant cells (strain MJL2807), which do not initiate recombination or produce SC [59], [60]. Bud emergence and nuclear divisions occurred at times similar to those seen in *SPO11* cells (Figure 1b), indicating that the extended gap phase seen upon RTG is not caused by a need to resolve recombination-dependent meiotic chromosome structures.

### The SC rapidly breaks down after RTG

*ndt80*Δ cells arrest with chromosomes that are fully paired by SC [49]. It was previously shown that the SC formed in *NDT80* cells breaks down rapidly after RTG [56]. We confirmed this observation in *ndt80Δ* strains by staining surface-spread nuclei for Zip1, a central component of the SC [61]. Most cells lose full-length linear SC within 15 minutes of transfer to YPD, and less than 30% of cells contained even residual (dotty) Zip1-containing structures 1.5 hr after RTG, before bud emergence and well before nuclear division (Figure 1c, 1d).

### Sister chromatids segregate during the nuclear division after RTG

**Figure 2:**
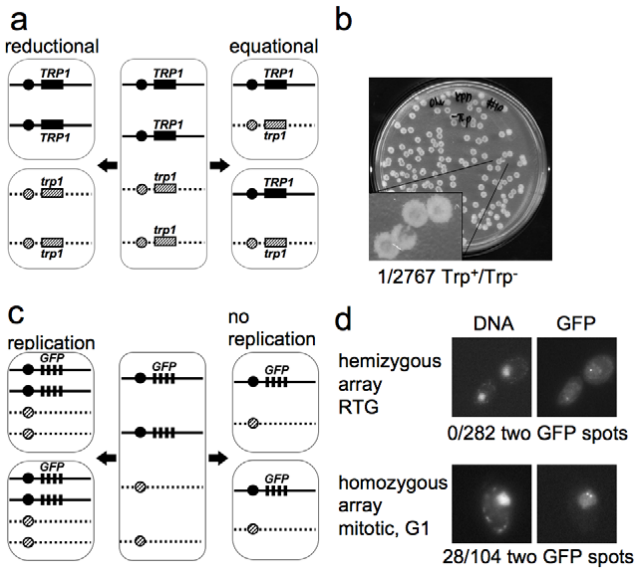
The first cell division after RTG involves equational chromosome segregation without replication. a. Outcome of different types of chromosome segregation after RTG. One homolog is shown as solid line and the other as dashed line. Black and diagonal hatched boxes indicate dominant *TRP1* and recessive *trp1* alleles, respectively. Reductional chromosome segregation (left) separates homologs, producing a sectored colony with *TRP1*/*TRP1* and *trp1*/*trp1* cells. Equational chromosome segregation (right) separates sister chromatids, producing homogenous *TRP1*/*trp1* colonies. b. Meiotic cells (MJL3163) were plated on YPD, inducing RTG, and 2767 colonies were replica-plated to medium lacking tryptophan. The single Trp^+^/Trp^−^ colony observed is shown. c. Expected outcomes if DNA replication occurs or does not occur before the first nuclear division after RTG. A strain hemizygous for a *CEN5-GFP* array (black rectangles, see text for details) is illustrated. After 7 hr in meiosis, each cell includes two copies of *CEN5-GFP* (middle). Replication followed by equational chromosome segregation (left) results in two copies of *CEN5-GFP* in each cell. Equational chromosome segregation without prior replication (right) leaves a single copy of *CEN5-GFP* in each cell. d. Upper panel—post-mitotic cells with a hemizygous *CEN5-GFP* array (MJL3312), from a sample taken 3.5 hr after RTG. All 282 post-mitotic G1 cells examined had a single GFP spot. Lower panel—control cells with a homozygous *CEN5-GFP* array (MJL3313) growing vegetatively in YPD. An unbudded cell in G1 is shown. 28/104 G1 cells had two GFP dots. Left—Nuclei detected by DNA/DAPI fluorescence; right—GFP fluorescence.

The first nuclear division of meiosis involves segregation of homologs (reductional division), whereas during mitotis, sister chromatids separate from each other (equational division). To determine if the first nuclear division after RTG is reductional or equational, we used a *TRP1*/*trp1* heterozygous strain. *TRP1* is tightly linked to the centromere of chromosome IV (<0.5cM; [62]), so chromosome segregation in the first division after RTG can be determined by examining *TRP1* allele segregation (Figure 2a). If the first division is reductional, one daughter cell will inherit both copies of the *TRP1* allele, whereas the other will inherit both copies of the *trp1* allele, resulting in a sectored Trp^+^/Trp^−^ colony. If the first division is equational, both daughter cells will inherit one *TRP1* and one *trp1* allele, resulting in a uniform Trp^+^ colony. A *TRP1/trp1 ndt80Δ/ndt80Δ* diploid (strain MJL3163) was induced to undergo meiosis for 7 hr, returned to growth by plating on YPD, and the resulting colonies were replica plated onto medium lacking tryptophan. Only one colony in 2767 was sectored, and the rest were uniformly Trp+ (Figure 2b). Thus, the first nuclear division after RTG involves a mitosis-like equational chromosome segregation.

### Cells do not replicate DNA before the first nuclear division after RTG

Because DNA replication can resolve JMs, it was important to determine whether or not cells undergo replication before the first division after RTG. During the mitotic cell cycle, bud emergence is closely followed by initiation of DNA replication [63]. We asked if bud emergence after RTG was also associated with DNA replication. *ndt80*Δ cells arrest after meiotic DNA replication, and thus have a 4C DNA content. Therefore, DNA re-replication before the first division after RTG will result in tetraploid daughter cells. On the other hand, if DNA re-replication does not occur after RTG, diploid daughter cells will be produced. To determine whether DNA re-replication occurs after RTG, we monitored the copy number of chromosome V, using a centromere-linked array of *tet* operator (*tetO*) repeats that bind a constitutively-expressed *tet* repressor-green fluorescent protein fusion [64], [65], referred to here as *CEN5-GFP*. To check the efficiency of detection of individual *CEN5-GFP* signals, diploids that were hemizygous (strain MJL3312) or homozygous (strain MJL3313) for *CEN5-GFP* were grown to log phase, and the number of GFP dots per nucleus was scored in unbudded cells (G1-phase of the cell cycle). As expected, unbudded cells with a hemizygous *CEN5-GFP* showed one dot per nucleus (133/133). In contrast, 28/104 unbudded cells homozygous for *CEN5-GFP* showed two dots in their nuclei (Figure 2d), indicating that two copies of *CEN5-GFP* are detected with about 25% efficiency. The reduced efficiency of detection of two GFP spots is most likely a result of the limited separation of centromeres during interphase in yeast, due to the close attachment of centromeres to the spindle pole body [66].

Using this assay, we determined the number of GFP dots in unbudded cells produced from the first or second division after RTG of a diploid with a hemizygous *CEN5-GFP* (strain MJL3312). Re-replication followed by an equational division would result in each daughter cell inheriting two copies of *CEN5-GFP*, and two GFP dots will be observed in the nucleus (Figure 2c). However, if no re-replication occurs, each daughter cell will inherit one copy of *CEN5-GFP*, resulting in one GFP dot in the nucleus. All cells examined (282/282) showed only one dot in each nucleus. Thus, cells do not undergo DNA replication before the first nuclear division after RTG.

To confirm the conclusion that cells do not undergo DNA replication before the first nuclear division after RTG, we monitored the copy number of the loosely centromere linked *MAT* locus. Re-replication, followed by an equational division, would result in most daughter cells being *MAT*a/*MAT*a/*MAT*α/*MAT*α tetraploids. However, if no re-replication occurs, most daughter cells will be *MAT*a/*MAT*α diploids. Sporulation of *MAT*a/*MAT*a/*MAT*α/*MAT*α tetraploid cells would frequently produce *MAT*a/*MAT*α nonmating diploid spores. On the other hand, sporulation of *MAT*a/*MAT*α diploid cells will only produce haploid spores with a single *MAT*a or *MAT*α allele (Figure S1).

To sporulate cells that are phenotypically Ndt80^−^, we used a strain (strain MJL3430, *pGPD1-GAL4-ER pGAL1-NDT80*; [67], [68], [10]) where *NDT80* is normally not expressed, but where *NDT80* expression can be induced by the addition of estradiol (ED). Seven independent segregants from RTG performed without *NDT80* expression (without ED) were induced to undergo a second meiosis with *NDT80* expression (with ED), and tetrads produced by these strains were dissected. All spores from 4 spore-viable tetrads (at least 10 tetrads per primary segregant; n = 400) were either *MAT*a or *MAT*α maters, and none were *MAT*a*/MAT*α nonmaters, confirming the conclusion that re-replication does not occur before the first nuclear division after RTG.

### Genetic evidence that COs are infrequently produced after RTG

**Figure 3:**
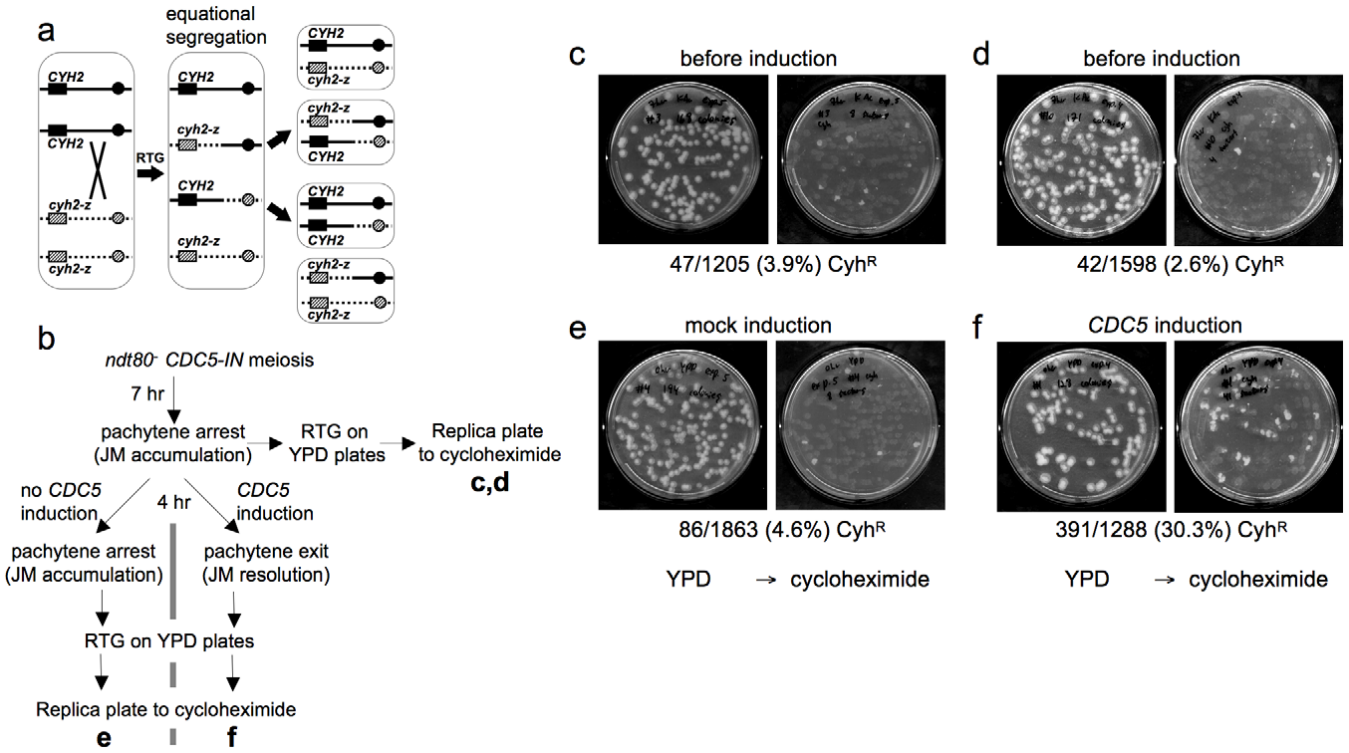
Few COs are produced after RTG. a. CO detection after RTG. Chromosome VII homologs are shown as solid and dashed lines. Black and grey boxes indicate dominant *CYH2* and recessive *cyh2-z* cycloheximide sensitive and resistant alleles, respectively. If a CO occurs between *CYH2* and the centromere, equational chromosome segregation produces either a colony that is uniformly *CYH2/cyh2-z* (cycloheximide-sensitive), or a colony with a *CYH2/CYH2* (cycloheximide-sensitive) sector and a *cyh2-z/cyh2-z* (cycloheximide-resistant) sector. b. Experimental design. *ndt80*Δ *CDC5-IN* (MJL3267) cells are incubated in sporulation medium for 7 hr to uniform pachytene arrest, and aliquots are plated on YPD for RTG (c and d). The culture is then incubated for an additional 4 hr without *CDC5* induction and plated on YPD (e), or the culture is incubated for 4hr in the presence of estradiol to induce *CDC5* expression before plating on YPD (f). Colonies on YPD are replica-plated to YPD + cycloheximide to detect *cyh2-z/cyh2-z* recombinants. c, d. Control aliquots plated directly on YPD before replica-plating to YPD + cycloheximide. e. Pachytene-arrested cells were incubated for 4 hr without *CDC5* induction before plating on YPD. f. Pachytene-arrested cells were incubated for 4 hr with estradiol to induce *CDC5* expression before plating on YPD. Note the marked increase in the frequency of cycloheximide-resistant segregants.

Since unresolved JMs are expected to interfere with chromosome segregation at mitosis, the observation that most *ndt80* mutant cells retain viability after RTG ([49]; see above) suggests that meiotic JMs must be resolved before the first cell division after RTG. During meiosis, JMs are mainly resolved to produce COs [8]–[10]. To ask if JMs are resolved similarly after RTG, we monitored segregation of the recessive cycloheximide–resistance allele, *cyh2-z*, in a *cyh2-z/CYH2* heterozygous diploid. In wild-type meiosis, 66% of cells undergo second division segregation for *cyh2-z*, resulting from crossing over between the *CYH2* locus and the centromere of chromosome VII (*CEN7*; see Materials and Methods). If JMs are similarly resolved as COs during RTG, 66% of cells are expected to have a CO between *CYH2* and *CEN7*. Assuming random sister chromatid segregation at the first division after RTG, as it is in mitosis [69], half of the cells with a CO between *CEN7* and *CYH2* will produce cycloheximide-resistant *cyh2-z*/*cyh2-z* daughter cells (33% of total colonies; Figure 3a).

To directly compare JM resolution after RTG and during meiosis, we used an *ndt80*Δ/*ndt80*Δ *CYH2/cyh2-z* strain that contains an estrogen-inducible *CDC5* gene (*ndt80*Δ *pGPD1-GAL4-ER pGAL1-CDC5*; strain MJL3267), to allow conditional JM resolution [10]. In the absence of inducer (-ED), cells accumulate in pachytene with unresolved JMs. ED addition induces *CDC5* expression, and cells exit from pachytene and resolve JMs to produce COs, but do not progress further through meiosis [10]. Thus, if *CDC5* is expressed before RTG, JMs will be resolved and COs will be produced at a level similar to that seen in meiosis. Thus, 33% of colonies are expected to be cycloheximide resistant (Figure 3a). Cells were induced to undergo meiosis for 7 hr, and then aliquots were plated on YPD to undergo RTG (Figure 3b). The remainder of the culture was incubated for another 4 hr in sporulation medium, either with ED to induce pachytene exit, or in the absence of ED as a control, and aliquots were plated on YPD. Colonies on YPD were replica plated onto YPD with cycloheximide to score for sectored colonies produced by crossovers. Only a small fraction of the RTG colonies from samples taken before mock or *CDC5* induction contained cycloheximide-resistant sectors (3.9% and 2.6%, respectively, Figure 3c, 3d), and cells plated after a 4 hr incubation without ED also produced few cycloheximide-resistant sectors (4.6%, Figure 3e). In contrast, when *CDC5* was expressed and JMs resolved as COs, 30% of colonies contained cycloheximide-resistant sectors (Figure 3f). The relatively low frequencies of colonies with cycloheximide-resistant sectors in all samples that underwent RTG without *CDC5* induction indicates that the majority of JMs are not resolved as COs after RTG.

### Molecular evidence that most JMs are not resolved as COs after RTG

**Figure 4:**
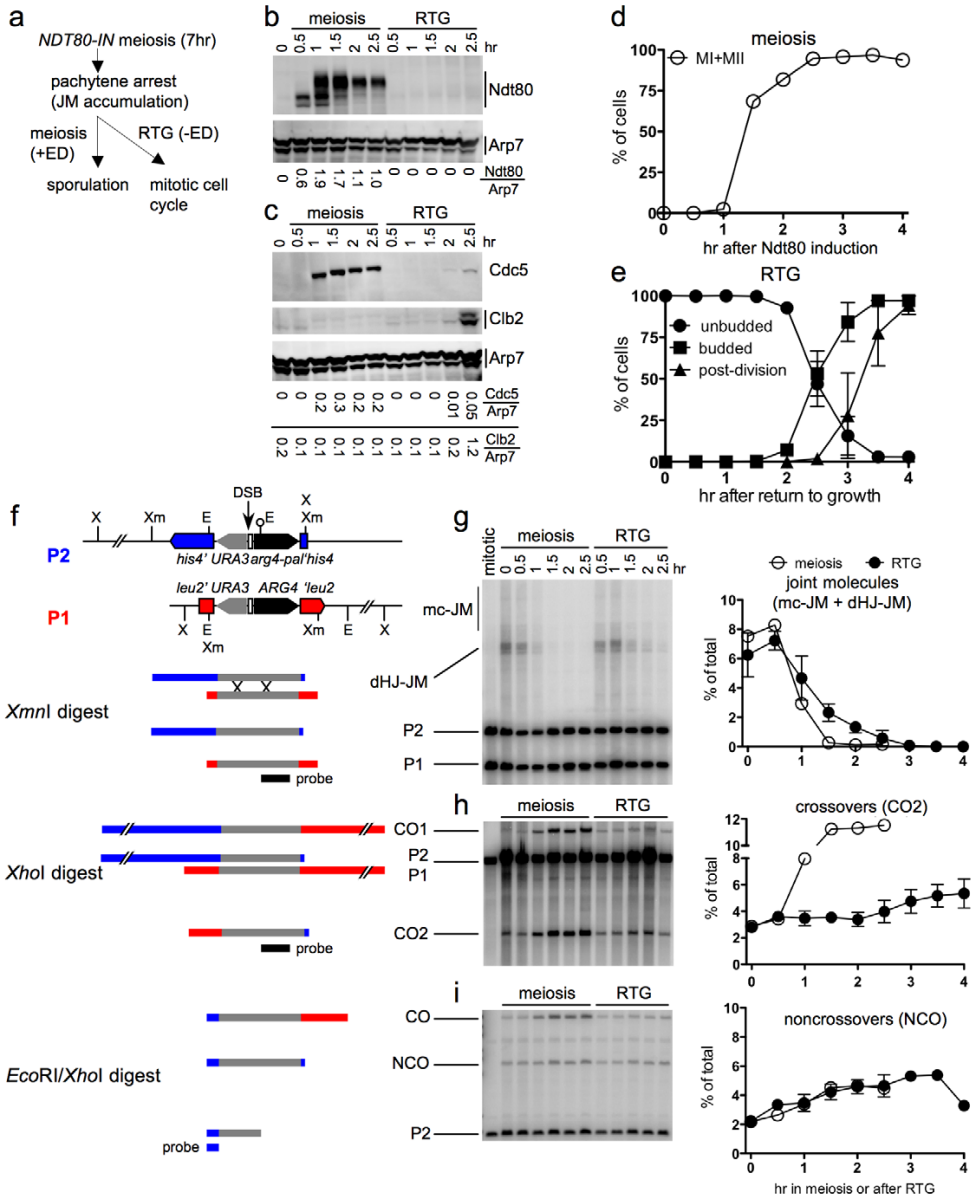
JM resolution and recombinant product formation during meiosis and RTG. a. Experimental design. Cells with an estrogen-inducible *NDT80* allele (MJL3430) are incubated in sporulation medium for 7 hr to uniform pachytene arrest. Estradiol (ED) is added to half of the culture to induce *NDT80* expression and the completion of meiosis, while the other half is transferred to YPD to undergo RTG in the absence of *NDT80* expression. b. Western blot showing Ndt80 production after addition of ED (meiosis) or after RTG. Arp7 is used as a loading control. Relative Ndt80 levels (arbitrary units) are shown below each lane. c. Western blot showing production of Ndt80-regulated polo-like kinase, Cdc5, and of the G2/M cyclin, Clb2, which is not expressed during meiosis. Arp7 is used as loading control. Relative protein levels (arbitrary units) are shown below each lane. d. Meiotic progression after *NDT80* induction by ED addition. The percentage of cells completing meiosis I in a single experiment was determined by DAPI staining and counting the fraction of cells with more than one nucleus (MI + MII). Values are from a single experiment. e. Cell cycle progression after RTG. Cell cycle events were scored as in Figure 1. Values are from three independent experiments. f. Recombination reporter system used to detect recombination intermediates and products [7]. A 3.5 Kb insert with the *URA3* (grey) and *ARG4* (black) genes is inserted at *LEU2* (red) on one chromosome III homolog and at *HIS4* (blue), 16.7 Kb away, on the other. 65 nt of yeast telomere sequences (open box), inserted between *URA3* and *ARG4*, create a strong meiotic DSB site (vertical arrow). A short palindrome containing an *Eco*RI site (lollipop) ∼0.6 kb from the DSB site, creates the *arg4-pal* allele in the insert at *his4*. Arrows denote the direction of transcription. Restrictions sites: Xm—*Xmn*I; X—*Xho*I; E—*Eco*RI. An *Xmn*I digest probed with *ARG4* sequences (black bar) detects dHJ-JMs. A *Xho*I digest probed with the same sequences detects CO products. An *Eco*RI/*Xho*I double digest, probed with *HIS4* sequences (blue bar) detects NCO events where the *arg4-pal* allele is converted to *ARG4* (full conversion shown), as well as a subset of COs (CO). It should be noted that a subset of NCOs are detected by this assay. Based on tetrad data from similar strains [7], we estimate that about 1/6 of total NCOs are detected. g–i. DNA was prepared from *NDT80-IN* cells (MJL3430) that were either induced to complete meiosis by ED addition or shifted to YPD to undergo RTG, as illustrated in a. Samples were analyzed for JMs, COs and NCOs as illustrated in f. Values for meiosis are from a single experiment; values for RTG are from three independent experiments (for JMs and COs) and two independent experiments for NCOs. g. JM intermediates. Left: blots of *Xmn*I digests probed with *ARG4* sequences. In addition to dHJ-JMs, JMs containing 3 or 4 chromatids (multichromatid, mc-JMs) were detected at low levels. Right: frequencies of all JMs, plotted as a percent of total lane signal. h. COs. Left: blots of *Xho*I digests probed with *ARG4* sequences. Right: CO product 2 (CO2) plotted as a percent of total lane signal. i. Noncrossover recombinants. Left: blots of *Xho*I*/Eco*RI digests probed with *HIS4* sequences. Right: NCOs, plotted as a percent of total lane signal.

Reduced CO formation after RTG was confirmed by molecular analysis. To allow direct comparison between events that occur during meiosis and during RTG, we used a recombination-reporter strain, described below, that also contained the estrogen-inducible *NDT80* allele described above (strain MJL3430) that confers reversible pachytene arrest [68]. Pachytene-arrested cells can be transferred to YPD without estradiol addition to undergo RTG in the absence of *NDT80* expression. Alternatively, they can be kept in sporulation medium, and by adding ED to induce *NDT80* expression, be made to complete meiosis (Figure 4a, 4b). Meiotic *NDT80* expression resulted in meiotic divisions (Figure 4d), spore formation (data not shown), and the rapid expression of *CDC5*, a known target of Ndt80 [70]. Cdc5 was detected one hr after addition of ED to meiotic cultures, whereas Cdc5 was not present in RTG cultures until 2–2.5 hr after the shift to YPD, about 30 min before nuclear division (Figure 4c, 4e). The mitotic cyclin Clb2, which is not produced during meiosis [71], was observed only in the RTG culture, at about the same time as Cdc5 (Figure 4c).

Recombination intermediate resolution and recombinant product formation were monitored at the molecular level, using a recombination reporter system [7] (Figure 4f). JM resolution initiated at similar times in both ED-induced meiotic and RTG cultures (Figure 4g). However, the two cultures differed markedly in terms of CO production. JM resolution in the meiotic culture was accompanied by a marked increase in crossovers in the same time interval, and was complete by 1.5 hr after Ndt80 induction (Figure 4h). In contrast, no increase in COs was seen in the first 2 hr after RTG, during which JMs decreased by five-fold. After two hr, a time that corresponded to the time of bud emergence (Figure 4e), resolution of the remaining JMs was accompanied by a modest increase in COs (Figure 4h). NCO products were produced in meiotic and in RTG cultures at similar levels (Figure 4i). Similar results were observed in RTG experiments using *ndt80*Δ cells lacking the inducible *NDT80* system (strain MJL3164; Figure S2).

The data presented here support the conclusion from genetic experiments described above, that most JMs are resolved after RTG without producing COs. The CO increase seen after 2 hr indicates that surviving JMs can be resolved as COs during the later stages of RTG.

### Efficient JM resolution without CO production after RTG in the absence of Mus81

**Figure 5:**
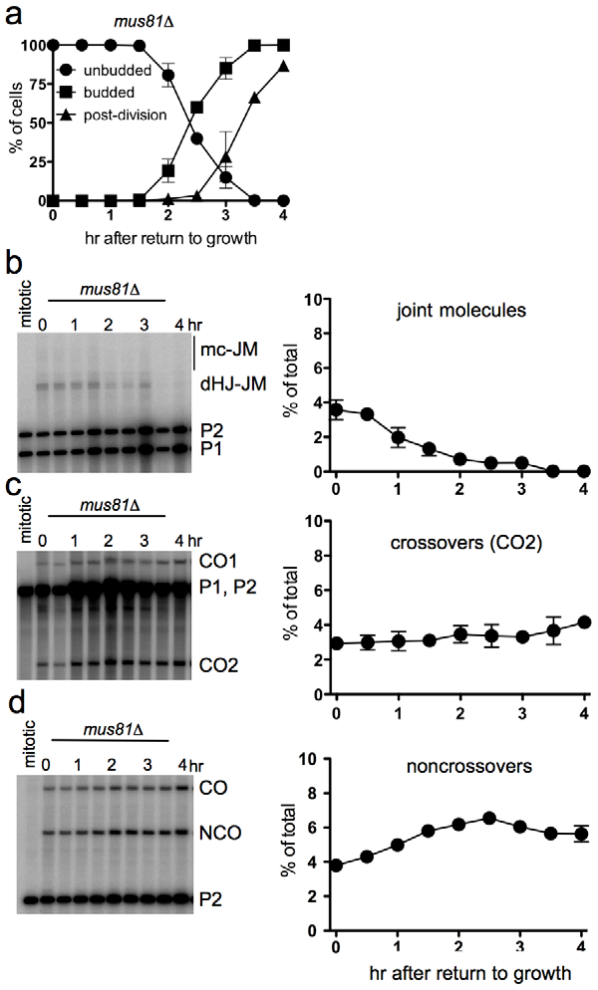
Efficient JM resolution without CO production after RTG in the absence of Mus81. a. Cell cycle progression of *ndt80*Δ *mus81*Δ cells (MJL3389) after RTG. Cell cycle events were scored as in Figure 1. b. JM intermediates. Left: blot of *Xmn*I digests probed with *ARG4* sequences as in Figure 4. Right: total JMs plotted as a percentage of total lane signal. c. COs. Left: blot of *Xho*I digests probed with *ARG4* sequences, as in Figure 4. Right: CO product 2 (CO2), plotted as a percentage of total lane signal. d. NCOs. Left: blots of *Xho*I*/Eco*RI digests probed with *HIS4* sequences, as in Figure 4. Right: NCO products plotted as a percentage of total lane signal.

The Mus81 complex plays a major role in JM resolution during meiosis in *S. pombe* and a less prominent role in meiotic JM metabolism in *S. cerevisiae* [19], [26], [20], [27], [22], [24], [72], [28]. To determine if the Mus81 complex resolves JMs after RTG, *ndt80*Δ *mus81*Δ cells (strain MJL3389) were induced to undergo meiosis for 7 hr and then transferred to YPD. Bud emergence and nuclear division occurred at times similar to those seen in *ndt80Δ MUS81* cells (Figure 5a, compare to Figure 1b). JMs were resolved completely after RTG (Figure 5b). A modest net increase in noncrossovers was seen (Figure 5d), similar to that seen in *MUS81* cells (see Figure 4i). Unlike in wild-type, where JM resolution after two hr was accompanied by an increase in COs, no significant CO increase was observed after RTG in *mus81Δ* mutants (Figure 5c). These data indicate that the Mus81 complex is not required for JM resolution after RTG, but it may play an important role in the limited JM resolution as COs that occurs at later stages.

### Delayed JM resolution after RTG in the absence of Sgs1 helicase activity

**Figure 6:**
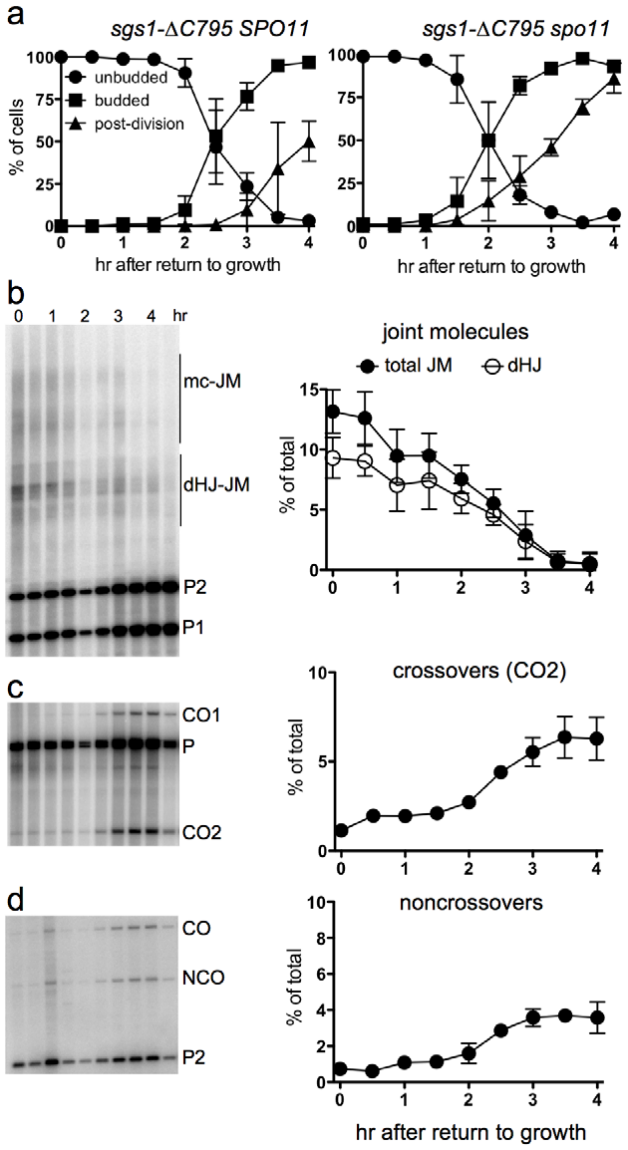
Delayed JM resolution and increased CO formation after RTG in the absence of the Sgs1 helicase. a. Delayed nuclear division during RTG of in the absence of Sgs1 helicase activity is due to meiotic recombination. Panels show cell cycle progression of *ndt80Δ sgs1-ΔC795* cells that are meiotic recombination competent (*SPO11*, left; MJL3388) or recombination null (*spo11*, right; MJL3428). b. Joint molecule intermediates. Left: blots of *Xmn*I digests probed with *ARG4* sequences. Right: frequencies of total JMs (multichromatid JMs, mcJMs plus dHJ-JMs, filled circles) and of dHJ intermediates (dHJ; empty circles) plotted as a percentage of total lane signal. c. Crossovers. Left: blots of *Xho*I digests probed with *ARG4* sequences. Right: CO product 2 (CO2) are plotted as a percentage of total lane signal. d. Noncrossovers. Left: blots of *Xho*I*/Eco*RI digests probed with *HIS4* sequences. Right: NCOs, plotted as a percentage of total lane signal.

The BLM and Sgs1 helicases, in combination with topoisomerase III and Rmi1/BLAP45, resolve dHJs *in vitro* as NCOs [33], [36]. To ask if Sgs1 has a similar role in JM resolution after RTG, we used an *sgs1* mutant allele (strain MJL3388; *sgs1*-Δ*C795*) that expresses only the first 652 amino acids of the protein [73], and which lacks both the helicase domain and a region (the HRDC domain) which in BLM interacts with Holliday junctions [74]. Although bud emergence occurred at a similar time after RTG in *sgs1-ΔC795* and in *SGS1* cells, nuclear division was 1.5–2 hr later in *sgs1-ΔC795* than in *SGS1* (Figure 6a, compare to Figure 1b). A recombination-null *ndt80*Δ *sgs1*-Δ*C795 spo11* triple mutant (strain MJL3428), which does not produce JMs, underwent nuclear division without this delay (Figure 6a), suggesting that the delay in nuclear division seen in *sgs1-ΔC795* might result from a delay in JM resolution.

To ask if JM resolution is delayed in *ndt80*Δ *sgs1*-Δ*C795* cells, we monitored JMs and recombination products, using the molecular assay system described above. As was previously described [41], *ndt80*Δ *sgs1*-Δ*C795* cells accumulate high levels of intersister JMs, and JMs with more than two chromatids (multi-chromatid JMs; mcJMs), in addition to the dHJ-JMs that accumulate in *ndt80Δ SGS1* cells (Figure 6b). Resolution of all JM species was delayed by about 1 hr in *sgs1-*Δ*C795* as compared to *SGS1*. While the vast majority of JMs resolved in *SGS1* by about 2.5 hr after RTG (Figure 4g), more than half of total JMs remained unresolved in *sgs1*-Δ*C795* at the same time, although all JMs resolved by 4 hr (Figure 6b). Thus, loss of the Sgs1 helicase results in a substantial delay in JM resolution after RTG.

Delayed JM resolution after RTG in *sgs1*-Δ*C795* was accompanied by altered recombinant product formation. COs increased only slightly in the first 1.5 hr after RTG (Figure 6c), but there was also only a slight increase in NCOs during the same period (Figure 6d). After 1.5 hr, JM resolution was accompanied by an increase in both COs and NCOs (Figure 6c, 6d). Thus, in both *SGS1* and in *sgs1*-Δ*C795*, few COs are produced during the first 1.5–2 hr after RTG, with substantially greater CO formation at later times. However, unlike in *SGS1*, where most NCOs appear in the first 1.5–2 hr after RTG, NCO production in *sgs1-ΔC795* is delayed until the time that COs also appear.

## Discussion

Most JM intermediates formed during budding yeast meiosis are produced by interhomolog recombination and are resolved as COs, and the majority of meiotic COs derive from interhomolog JMs [8], [9], [17], [10]. In contrast, interhomolog JMs and COs are less prominent during the mitotic cell cycle. Most JMs produced during mitotic DSB repair involve sister chromatids [15], and only a minor fraction (typically 5–10%) of mitotic recombination involves crossing-over, as would be expected if interhomolog JMs are rarely resolved as COs during the mitotic cell cycle [16], [75]. Testing this suggestion has, to date, been limited by the very low levels of interhomolog JMs produced in vegetatively-growing cells, even when initiating DSBs occur at levels similar to those seen in meiosis [15].

In this paper, we used RTG as an alternate approach to the study of JM resolution during the mitotic cell cycle. Although aspects of RTG have been examined in many studies [50]–[58], interpretation has been complicated by the relatively poor synchrony of yeast meiotic cultures. Thus, RTG samples from normal meiotic cultures can contain cells with unrepaired DSBs, cells with repaired DSBs but unresolved recombination intermediates, and cells where intermediates already have been resolved. To avoid complications inherent in the analysis of such a complex mixture, we performed RTG using meiotic cultures of *ndt80* mutant cells, which arrest at a single stage of meiosis (pachytene), with chromosomes fully paired by synaptonemal complex and with high levels of interhomolog JMs. This has provided insight into features of the mitosis-like cell cycle that immediately follows exit from meiosis, and into mechanisms of the recombination intermediate resolution.

### Return to growth involves a mitosis-like division without an intervening S-phase

When transferred from sporulation to growth medium, yeast cells degrade most meiotic transcripts within 20 min, and return to a pattern of gene expression that roughly resembles the G1 phase of the mitotic cell cycle [57]. Despite this rapid change in transcription patterns, cells spend an extended lag period (1.5 to 3 hours, equivalent to one or two normal mitotic cell cycles) before they undergo bud emergence, the first outward sign of resumed growth (Figure 1). Although cells disassemble synaptonemal complex and resolve meiotic recombination intermediates during this period ([56], this work), a similar lag before bud emergence is seen in *spo11* mutants (this work), and also if SC disassembly and JM resolution occur before RTG, by virtue of Cdc5 induction in *ndt80Δ CDC5-IN* cells (Y.D. and M.L., unpublished observations). It is therefore likely that this extended gap phase represents the time needed for metabolic adjustment to the shift from acetate to glucose, and from nitrogen-depleted to nitrogen-rich medium, rather than the time needed to disassemble meiosis-specific chromosome and DNA structures.

During the mitotic cell cycle, bud emergence is accompanied by the initiation of chromosome replication [63], but this is not the case during RTG. We used two different approaches to confirm that bud emergence occurs without DNA replication after RTG [53]. This could be the consequence of a failure to express completely the ensemble of proteins necessary for DNA replication. While some replication protein-encoding genes are transcribed after RTG ([57], Lea Jessop and M. L., unpublished observations), transcripts of *DBF4* and *CDC7*, which encode a kinase critical for replication origin firing, are rapidly reduced upon RTG [57]. Re-replication may also be blocked if cyclin-dependent kinase remains at post-S phase levels throughout RTG, which would prevent origin re-licensing [76]–[78].

We also find that the first nuclear division after RTG involves an equational division, unlike the reductional division that occurs during meiosis I. Reductional division at meiosis I requires the loading, at kinetochores, of the meiosis-specific protein complex monopolin, which promotes co-orientation of sister kinetochores towards a single spindle pole [79], [80]. Monopolin contains a meiosis-specific protein, Mam1, and two nucleolar proteins, Csm1 and Lrs4, whose kinetochore localization requires Cdc5 activity [79], . Meiotic *CDC5* transcription requires *NDT80*, and *MAM1* transcripts are reduced in *ndt80* mutants [70] and rapidly decline upon RTG [57]. In addition, monopolin loading at kinetochores requires active Cdc7/Dbf4 kinase [82], which is most likely not produced after RTG [57]. Therefore, it is unlikely that monopolin is loaded at kinetochores during RTG of *ndt80Δ* cells, and thus it is not surprising that the first nuclear division after RTG is equational.

### Recombination intermediate resolution during RTG is biased against crossovers

Most of the Holliday junction-containing JMs that accumulate during meiosis in *ndt80* mutants are resolved as COs upon restoration of either *NDT80* or *CDC5* gene expression ([10], this work). In contrast, our genetic and molecular analyses show that most of the JMs that form during wild-type meiosis are resolved without crossover formation during RTG. This indicates that mechanisms of JM resolution that operate during RTG differ from those that operate during meiosis.

**Figure 7:**
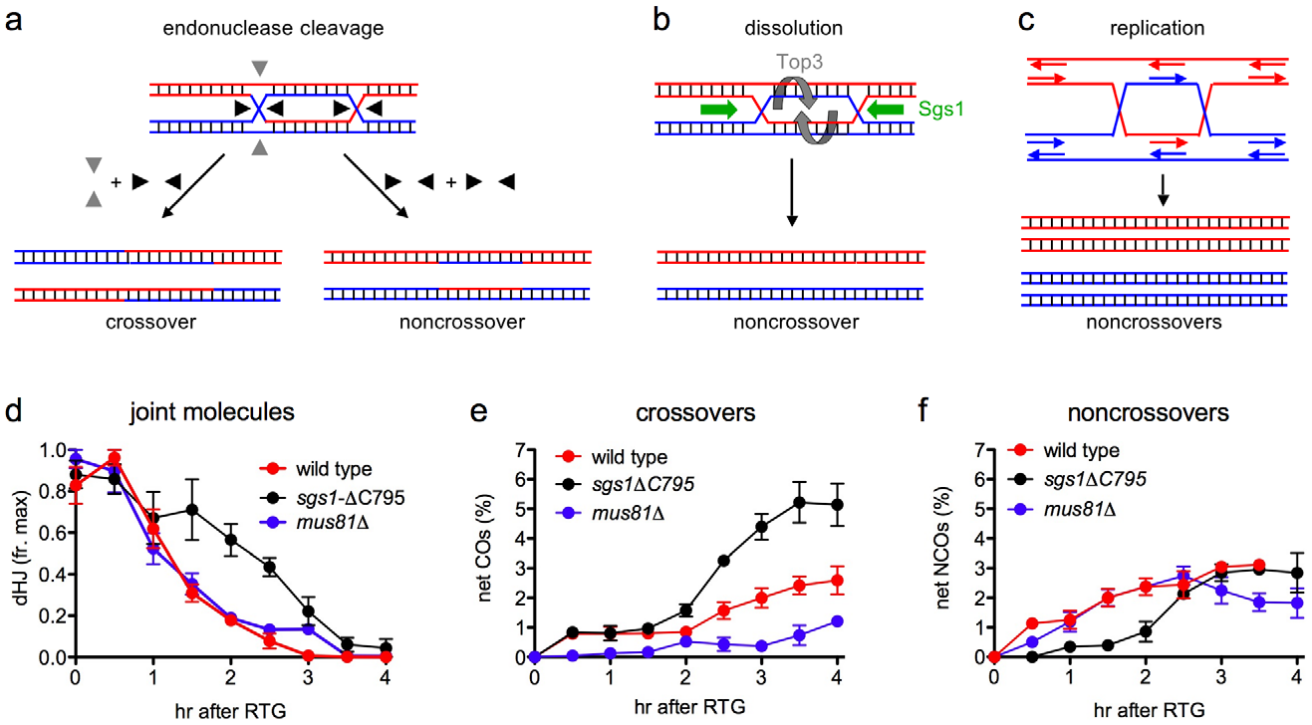
Modes of dHJ-JM resolution and summary of data. a. Resolution by junction cleavage [83]. Cleavage of both Holliday junctions in the same orientation (black arrows) yields noncrossovers; cleavage of the two junctions in orthogonal orientations (black and grey arrows) yields crossovers. For simplicity, only one of the two patterns for each type of cleavage is shown. b. Resolution by dissolution [31], [32]. Helicase-driven convergent junction branch migration, coupled with topoisomerase-removal of superhelical stress, produces only noncrossovers. c. Resolution by replication produces only noncrossovers. d. Summary of JM resolution during RTG. Maximum JM levels in each individual experiment (3 for wild-type, 2 for *sgs1-ΔC795* and *mus81Δ*) were set to 1. For *sgs1-ΔC795*, 2-chromatid JM values were used, although similar results are obtained with total JMs (2-chromatid + multichromatid). Plotted values represent averages; error bars indicate standard error of the mean. e. Net CO production during RTG. CO levels at 0 hr (the time of RTG) were subtracted from each time-point value and plotted as in d. f. Net NCO production during RTG. NCO levels at 0 hr (the time of RTG) were subtracted from each time-point value and plotted as in d.

There are three general mechanisms for dHJ-JM resolution: endonuclease cleavage; helicase/topoisomerase-mediated dissolution; and replication (Figure 7a–7c). Of these, replication and dissolution produce only NCO products, while endonuclease cleavage can, in principle, produce either COs or NCOs, depending upon the orientation of the two cleavage reactions. Since most dHJ-JMs resolve as COs during meiosis, meiotic resolution must involve endonuclease cleavage, and this cleavage must be constrained so that the two Holliday junctions are usually cut in opposite directions (see Figure 7a).

In contrast, JM resolution during RTG appears to occur in two phases with different outcomes (Figure 7d–7f). In wild-type cells, about 80% of JMs disappear during the first 1.5–2 hr after RTG. Few COs are produced during this period, and NCOs increase to near-final levels. The greatest net increase in COs occurs at 2 hr and later (Figure 7e), when the remaining 20% of JMs are resolved (Figure 7d). Thus, RTG appears contain an initial period (hereafter called early RTG) that precedes bud emergence, during which SC breaks down (Figure 1c) and the majority of JMs resolve without CO formation (Figure 7d, 7e). During the second period (hereafter called late RTG), between bud emergence and nuclear division, JM resolution is accompanied by CO formation.

### Sgs1-dependent dissolution as a mechanism for JM resolution during early RTG

JM resolution without CO formation, which predominates during early RTG, could occur by endonucleolytic cleavage that is constrained to produce only NCOs, by dissolution, or by replication (Figure 7a–7c). Resolution by replication is unlikely, since all available evidence indicates that the first cell division after RTG occurs without prior replication (this work, [53]). Both JM resolution and NCO formation are significantly reduced during early RTG in *sgs1-ΔC795* mutant cells (Figure 7d, 7f), which lack both the helicase and Holliday junction-binding domains of this RecQ helicase [73], [74]. The most parsimonious interpretation of these data is that, in wild-type cells, JM resolution during early RTG occurs primarily by dissolution, catalyzed by Sgs1 and Top3/Rmi1, as has been observed *in vitro* [36]. However, it is formally possible that other activities are responsible for the initial phase of JM resolution in wild-type, and that, unlike in wild-type, the majority JMs that form during *sgs1-ΔC795* meiosis have structures that are refractory to resolution by these hypothetical activities.

During budding yeast meiosis, the Sgs1 helicase acts with Mus81/Mms4 to prevent the accumulation of abnormal recombination intermediates [28], [29]. Normal JM intermediates are protected from Sgs1 by components of the synaptonemal complex, and *sgs1-ΔC795* partially suppresses the JM deficit observed in mutants lacking SC components [40], [42], [41]. These and other observations have been interpreted as indicating that Sgs1 acts primarily to prevent JM formation during meiosis. Our current data indicate that, in addition to preventing JM formation, Sgs1 can also dissolve JMs *in vivo*, but is prevented from doing so during meiosis by the SC. This suggestion is also supported by the finding that most JMs are resolved without CO production upon Cdc5-independent SC breakdown in pachytene-arrested meiotic cells (Anuradha Sourirajan, Arnaud de Muyt and M. L., unpublished observations).

### JM resolution by endonucleolytic cleavage during late RTG

While JM resolution during early RTG is rarely accompanied by CO production, JMs that survive this initial phase appear to be resolved frequently as COs. This is seen in wild-type, but is most evident in *sgs1-ΔC795* mutant cells, where an increase in the rate of JM resolution during late RTG is accompanied by a marked increase in both CO and NCO recombinants (Figure 7e, 7f). Because COs can only be produced by endonuclease-mediated JM cleavage, this suggests that a Holliday junction resolvase is activated 1.5–2 hr after RTG, a time that is also marked by bud emergence. We do not know the regulatory change that is responsible for this change in modes of JM resolution, but it is worth noting that both Cdc5 and the G2/M phase cyclin, Clb2, are first produced at this time (Figure 4c).

During meiosis, the Cdc5 kinase triggers JM resolution as COs [10], suggesting an obligate cleavage of JM Holliday junctions in opposite directions (Figure 7a). In contrast, JM resolution during late RTG of *sgs1-ΔC795* mutants produces both COs and NCOs (Figure 7e, 7f), as would be expected for the mixed parallel and opposite cleavage patterns contained in the original DSBR model ([83], see Figure 7a). This apparent difference in resolution mechanisms may reflect the chromosome environment in which intermediates reside. While JM resolution during late RTG occurs in the absence of detectable SC, crossover-designated meiotic JMs are thought to reside in SC-associated structures, called late recombination nodules, that contain the Holliday junction-binding proteins Msh4/Msh5 and associated Mlh1, Mlh3 and Exo1 proteins [84]–[86]. In *mlh1, mlh3*, and *exo1* mutants, meiotic JM levels are normal but crossover formation is reduced roughly two-fold [87], [88], consistent with the suggestion that the Mlh1/Mlh3/Exo1 components of late recombination nodules direct nuclease-mediated meiotic JM resolution towards a crossover-only outcome. In the absence of such specialized chromosome structures, nuclease-mediated JM resolution may be more evenly divided between COs and NCOs, in both mitotic and meiotic cells.

### A role for Mu81/Mms4 in JM resolution during RTG?

Although the nuclease(s) responsible for dHJ resolution during either meiosis or during RTG remain to be determined, it is worth noting that CO formation during RTG is even more reduced in *mus81Δ* mutants than in wild-type (Figure 7e), and the increase in COs seen during late RTG in wild-type and in *sgs1-ΔC795* is not seen in *mus81Δ* mutants. In many organisms, including *S. cerevisiae*, the Mus81 nuclease complex is dispensable for most meiotic COs [26], [89]–[91], and the majority of meiotic JMs resolve in a timely manner in *S. cerevisiae mus81* or *mms4* mutants [27], [28]. In addition, it has been reported that intact Holliday junctions are a relatively poor substrate for the Mus81/Mms4 nuclease, while junctions with one nicked strand are resolved efficiently [92], [22], [93]. On the other hand, *MUS81* is required for timely disappearance of X-shaped DNA molecules that form in methyl methanesulfonate-treated *rmi1-ts* cells [94]. This would suggest a role for Mus81/Mms4 in resolving these JMs, whose structure remains to be determined.

Our data suggest that Mus81/Mms4 has a role in resolving the JMs that survive until late RTG, but it does not appear to be active during early RTG. It is possible that either Mus81/Mms4 or a junction nicking activity that converts HJs into a Mus81/Mms4 substrate are absent during early RTG. Alternatively, the Mus81 complex may be modified during late RTG so that it resolves intact Holliday junctions unassisted. The latter suggestion, if correct, might explain the failure to observe robust Holliday junction resolution activity in most biochemical studies [95].

### Concluding remarks

In this work, we have shown that Holliday junction-containing recombination intermediates, formed during meiosis, are resolved during RTG in a manner that substantially reduces CO production. To the extent that recombination is regulated similarly during RTG and during the mitotic cell cycle, and to the extent that similar recombination intermediates are present, this finding can help explain the relatively low yield of COs during mitotic recombination. In particular, our findings reinforce the identification of the BLM family of RecQ helicases as playing an important role in suppressing CO recombination during the mitotic cell cycle [38]. Our findings also suggest that the Mus81 complex is the primary nuclease responsible for mitotic CO recombination [30]. Our finding, that these two enzymes act during different phases of the period before the first cell dvision after RTG, raises the intriguing possibility that the mitotic cell cycle may be similarly partitioned. It is attractive to suggest that helicase-mediated dissolution predominates during most of the mitotic cell cycle, with endonuclease-mediated JM cleavage being activated at the end. This would minimize the potential for CO-mediated loss of heterozygosity and chromosome entanglement, while preserving the ability to resolve JMs that escape dissolution before the initiation of mitosis.

In applying conclusions regarding JM resolution during RTG to the mitotic cell cycle, it should be kept in mind that these processes are not identical. For example, RTG involves the disassembly of chromosome structures that are not present during the mitotic cell cycle, as well as S-phase bypass, and both of these differences have the potential to affect modes of JM resolution. It will be of considerable interest to examine, during RTG, patterns of expression and modification of proteins involved in recombination, repair, and cell cycle progression during meiosis and the mitotic cell cycle.

## Materials and Methods

### Yeast strains and media

Strains are listed in Table S1 and are SK1 derivatives [96]. The *URA3*-*ARG4* recombination interval has been described [7]; *cyh2-z* is a spontaneous cycloheximide resistance mutation (Cyh^R^); *spo11-Y135F* [97] was a gift from S. Keeney; *mus81*Δ and *sgs1*-Δ*C795* have been described [42], [28]. Strains with estrogen-inducible *CDC5* and *NDT80* alleles (*pGPD1-GAL4-ER pGAL1-CDC5* and *pGPD1-GAL4-ER pGAL1-NDT80*, respectively) have been described [10]. Strains were constructed by genetic crosses, or by transformation. Media formulae were as described [98], [99].

### Liquid sporulation and return to growth

Sporulation was as described [99] using 400 ml cultures in a 2.8 liter baffled Fernbach flask (BellCo Glass) with a cell density of 2x 10^7^ cells per ml at the beginning of sporulation. For RTG experiments, cells were induced to undergo meiosis for 7 hr, harvested by centrifugation, resuspended in an equal volume of liquid YPD (prewarmed to 30°C) and aerated with vigorous shaking at 30°C in conditions similar to those used for sporulation. For plating experiments, samples were sonicated twice for 5 seconds at baseline power (Microson XL 2005), diluted appropriately and then plated on YPD plates. To determine colony-forming units, samples were counted in a hematocytometer and the concentration of cells was determined; cells with unseparated buds were counted as a single entity. For Ndt80 or Cdc5 induction, β-estradiol (ED; Sigma; 5 mM stock in ethanol) was added to a final concentration of 1 µM. For no Cdc5-indcuation control experiments, the same amount of ethanol (without ED) was added. For RTG after Cdc5 induction during meiosis, cells were washed twice with sporulation medium lacking ED at 30°C before resuspension in YPD.

Unless stated otherwise, all data presented are the average of two independent experiments; error bars in plots indicate standard error.

### Cytology

To score bud emergence and nuclear division, 1 ml of a culture was mixed with 1 ml of ethanol and stored at 4°C. Just before examination, 1 µl of 1 mg/ml 4′,6-diamidino-2-phenylindole (DAPI) was added and samples were left for 5 min at room temperature, washed once with an equal volume of water and resuspended in 0.5 ml water. Cell morphology was scored using phase contrast or differential interference contrast microscopy and nuclear morphology by DAPI epifluorescence microscopy, using a Zeiss Axioplan 2 epifluorescence microscope and a QICAM camera. Images were acquired using QCapture 3.1.1 and processed with Adobe Photoshop CS3.

GFP chromosome dot visualization was done using cells fixed in 3.7% formaldehyde as described [65]. Vectashield with DAPI (Vector Laboratories) was used to simultaneously stain DNA. Cells were counted as having two GFP dots if two separated GFP dots could be clearly visualized. Sample fluorescence was visualized using a Zeiss Axioplan 2 epifluorescence microscope and a Micromax 1300 CCD camera. Images were acquired using IPlab 3.7 and processed with Adobe Photoshop CS3.

Nuclear spreads were performed and stained as described [100] using cells from 5 ml of culture. Zip1 was detected using anti-Zip1 rabbit polyclonal sera (a gift from G.S. Roeder, 1∶100 dilution) as the primary antibody and Alexafluor 488 conjugated goat anti-rabbit IgG (Molecular Probes #A11034) at 1∶100 as the secondary antibody. To visualize DNA, 40 µl of Vectashield with DAPI (Vector Laboratories) was added. Sample fluorescence was visualized using a Zeiss Axioplan 2 epifluorescence microscope and a Micromax 1300 CCD camera. Images were acquired using IPlab 3.7 and processed with Adobe Photoshop CS3.

### Calculation of cumulative curves for bud emergence and nuclear division

During RTG, cells lose synchrony and continue to further cell cycles, complicating calculation of a cumulative cell division curve. We assumed that bud emergence and nuclear division occur with the same relative timing in the first and second cell division after RTG. To distinguish between daughter and mother cells, we took advantage of the fact that after RTG, *ndt80*Δ cells produce an elongated bud that can be easily distinguished from the round mother cell (Figure 1a). The fraction of cells that had not yet budded (unbudded cells) was calculated according to the equation: unbudded cells =  (X_1_-Y_1_)/Z_1_ where X_1_ =  unbudded round cells (i.e. cells before the first mitotic division), Y_1_ =  unbudded elongated cells (i.e. products of the first mitotic division) and Z_1_ =  total cells counted. At late times, due to continuous division of the cells, the number of cells that have already undergone the first mitotic division (Y_1_) can exceed the number of cells that have not undergone a mitotic division (X_1_). In such a case, (X_1_-Y_1_) was set to zero.

The fraction of cells that had undergone the first nuclear division (post-division) was calculated according to the equation: post-division =  X_2_/Y_2_ where X_2_ =  round cells that were undergoing mitosis (detected as budded with a nucleus stretched between the mother and daughter cells) plus all elongated cells with a nucleus (i.e. cells that have already completed the first mitotic division) and Y_2_ =  all round cells. At late times, due to continuous cell division, X_2_ may be greater than Y_2_. In such a case, the fraction of post-division cells was set to one.

### DNA extraction and digestion

DNA preparation and analysis on Southern blots were as described [101], [8]. *Xho*I and *Xmn*I digests were probed with *ARG4* coding sequences (+165 to +1413). XhoI/*Eco*RI double digests were probed with *HIS4* coding sequences (+538 to +718).

### Protein analysis

Protein was prepared from 4 ml of sporulating culture by TCA precipitation [102]. 5 µl samples of each extract were displayed on 7.5% polyacrylamide Tris-Glycine pre-cast gels (Bio-Rad) and electroblotted to a PVDF membrane (Invitrogen), using an iBlot Dry Blotting System (Invitrogen) as recommended by the manufacturer. Blots were washed for at least one hr on an orbital shaker at room temperature in blocking buffer, 0.2% I-block (Tropix) in PBST (0.15 M NaCl, 0.053 M Na_2_HPO_4_, 0.008 M KH_2_PO_4_, 0.05% v/v Tween-20, pH 7.4). Primary antibody, diluted in blocking buffer, was added to the blot and incubated on an orbital shaker at room temperature for at least one hr. Blots were washed four times for 15 min with blocking buffer, incubated with secondary antibody for one hr with shaking at room temperature, and wash steps were repeated. Signal was developed using the chemiluminescent CDP-star substrate (Applied Biosystems), detected using a Fuji LAS3000 CCD camera, and quantified using ImageGauge V4.22 software (Fuji). Blots were stripped with OneMinute Western Blot Stripping Buffer (GM Biosciences) and reprobed for Arp7 as a loading control. Primary antisera were as follows: Arp7 – goat polyclonal (Santa Cruz Biotechnology, Inc; Sc-8961), 1∶500; influenza hemagglutinin (HA) – mouse monoclonal (5 µg/µl; Roche Applied Science; 12CA5), 1∶10,000; Cdc5 – goat polyclonal (Santa Cruz Biotechnology, Inc; Sc-6733), 1∶500; Ndt80 – rabbit polyclonal (a gift from K. Benjamin), 1∶10,000; Clb2 – rabbit polyclonal (Santa Cruz Biotechnology, Inc; Sc-9071), 1∶500. Secondary antibodies were alkaline phosphatase conjugates of goat-anti-mouse (Sigma, A3562), goat-anti-rabbit (Sigma, A3687) and rabbit-anti-goat (Sigma, A4187), all used at 1∶10,000.

### Measuring crossovers between *CYH2* and the centromere

To measure the frequency of recombination between the *CYH2* locus and the centromere of chromosome VII, we measured second division segregation pattern of the *TRP1* and *CYH2* alleles in dissected tetrads from strain MJL3548 (*CYH2*/*cyh2-z TRP1*/*trp1*), using *TRP1* as a centromere-linked marker [62]. Of 72 tetrads with 4 viable spores, 12 tetrads were parental ditypes, 12 were non-parental ditypes and 47 were tetratypes. One tetrad had gene conversion of *cyh2-z* and was not counted. Thus, as expected for a locus far removed from its centromere, the vast majority of cells undergo at least one crossover between *CYH2* and *CEN7*, and about two thirds of cells produce spores with a crossover between the *CYH2* locus and its centromere.

## Supporting Information

Figure S1 Expected outcomes if DNA replication occurs (a) or does not occur (b) before the first nuclear division after RTG. One homolog is shown as solid line and the other as dashed line. Black and diagonal hatched boxes indicate *MAT*a and *MATα* alleles, respectively. After 7 hr in meiosis (left in a and b), each cell contains two copies of each *MAT* allele. a. Replication followed by equational chromosome segregation results in two copies of each *MAT* allele in each daughter cell. Sporulation of these cells produces *MAT*a/*MATα* nonmater, *MAT*a/*MAT*a mater and *MATα*/*MATα* mater diploid cells. b. Equational chromosome segregation without prior replication leaves one copy of each allele. Sporulation of these cells produces only haploid mater cells. See text for details.
(TIF)

Figure S2 JM resolution after RTG in an *ndt80Δ* diploid cells (MJL3164). After 7 hr in sporulation medium, cells were shifted to YPD to undergo RTG. 0 hr – time of shift to YPD. See Figure 4 for digest and probe details. a. JM intermediates. Left: blots of *Xmn*I digests probed with *ARG4* sequences. Right: JM frequencies, plotted as a percent of total lane signal. b. COs. Left: blots of *Xho*I digests probed with *ARG4* sequences. Right: CO2 frequencies plotted as a percent of total lane signal. c. NCOs. Left: blots of *Xho*I*/Eco*RI digests probed with *HIS4* sequences. Right: NCO frequencies plotted as a percent of total lane signal.
(TIF)

Table S1 Strains used in this work. All are *MAT* **a**/*MATα lys2/lys2 ho::LYS2/ho::LYS2*. The *ndt80* allele is *ndt80*Δ*(Eco47III-BseRI)::KanMX6*. MJL2984-derived strains contain the recombination reporter illustrated in Figure 5.
(DOC)
