# Supplementary figures and images for: Meiotic Recombination Intermediates Are Resolved with Minimal Crossover Formation during Return-to-Growth, an Analogue of the Mitotic Cell Cycle
Source: PLoS Genet. 2011 May 26;7(5):e1002083. doi: 10.1371/journal.pgen.1002083 (PMC3102748; doi:10.1371/journal.pgen.1002083)

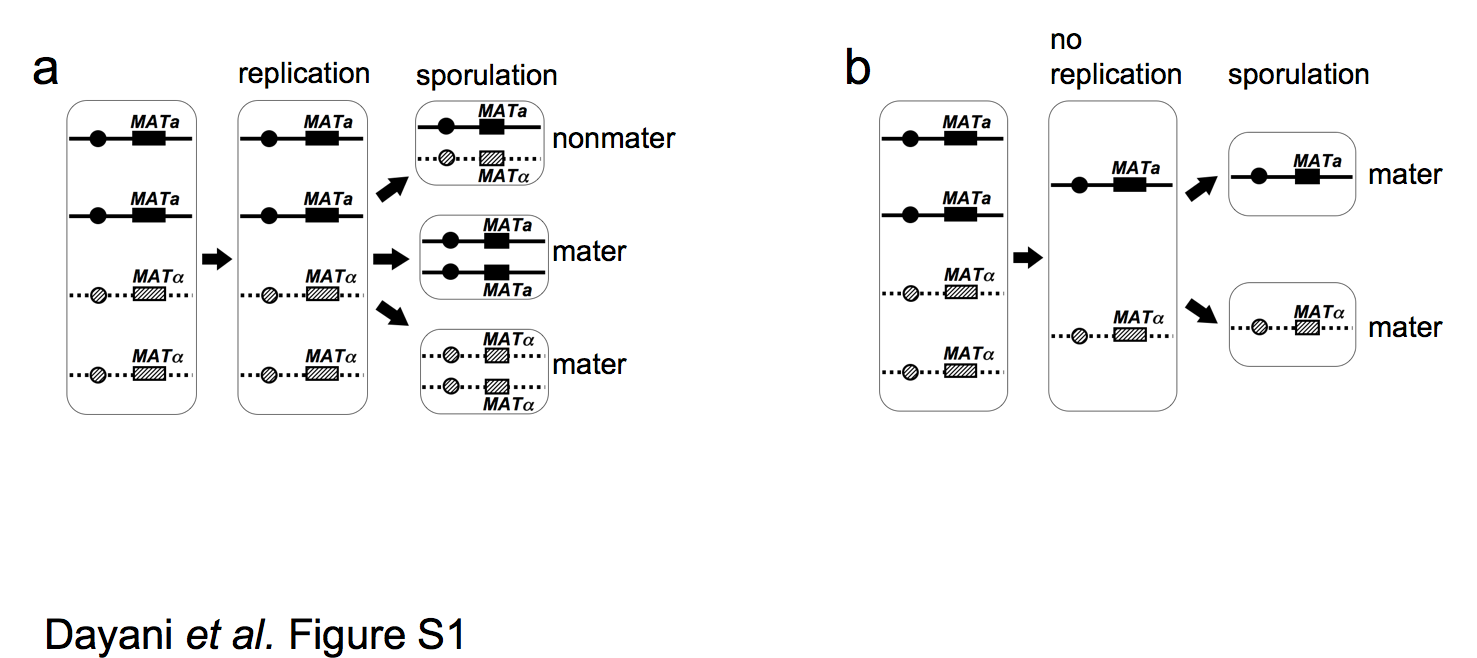

Supplement: Figure S1 — Expected outcomes if DNA replication occurs (a) or does not occur (b) before the first nuclear division after RTG. One homolog is shown as solid line and the other as dashed line. Black and diagonal hatched boxes indicate MATa and MATα alleles, respectively. After 7 hr in meiosis (left in a and b), each cell contains two copies of each MAT allele. a. Replication followed by equational chromosome segregation results in two copies of each MAT allele in each daughter cell. Sporulation of these cells produces MATa/MATα nonmater, MATa/MATa mater and MATα/MATα mater diploid cells. b. Equational chromosome segregation without prior replication leaves one copy of each allele. Sporulation of these cells produces only haploid mater cells. See text for details. (TIF) [file pgen.1002083.s001.tif]

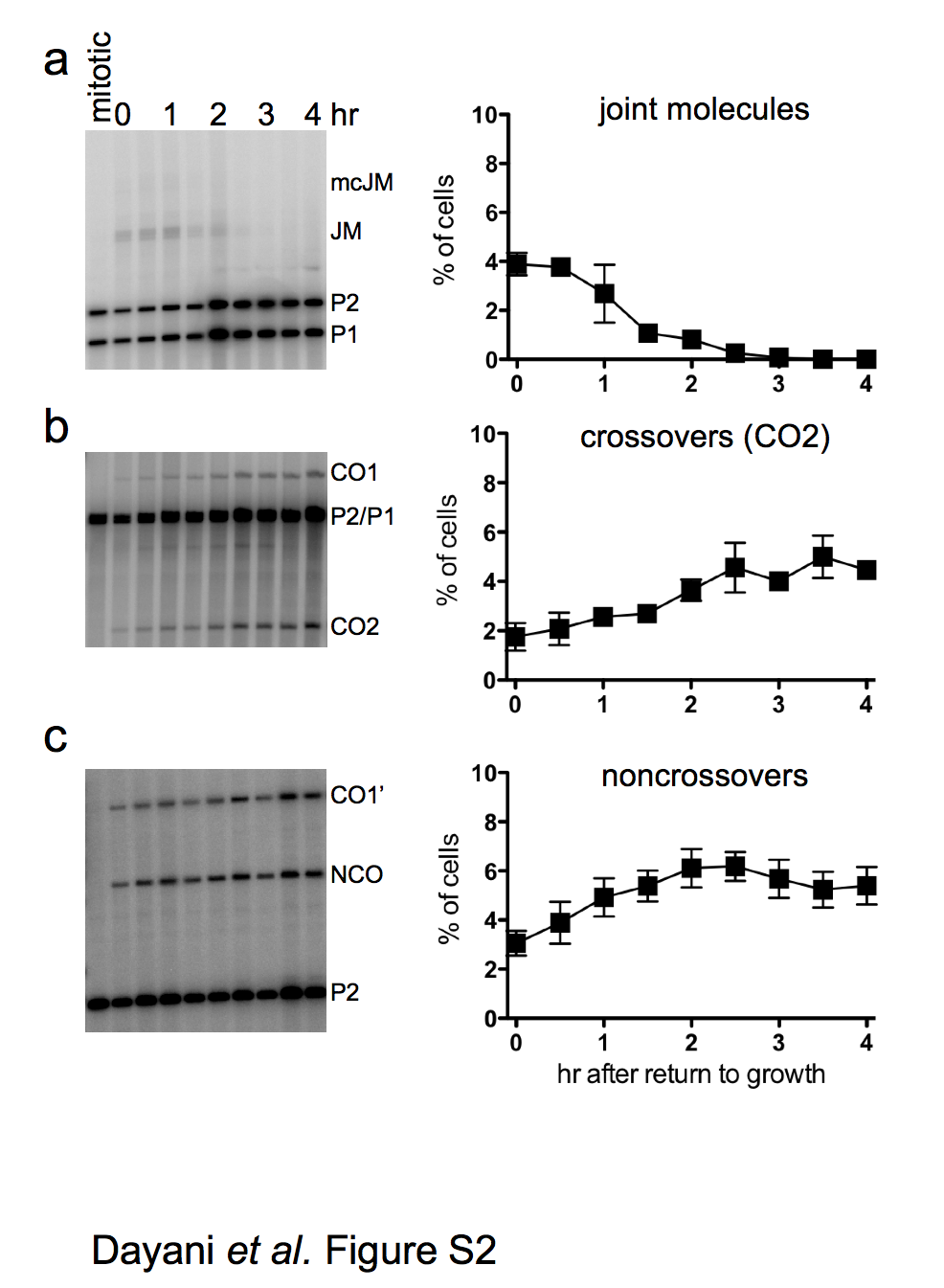

Supplement: Figure S2 — JM resolution after RTG in an ndt80Δ diploid cells (MJL3164). After 7 hr in sporulation medium, cells were shifted to YPD to undergo RTG. 0 hr – time of shift to YPD. See Figure 4 for digest and probe details. a. JM intermediates. Left: blots of XmnI digests probed with ARG4 sequences. Right: JM frequencies, plotted as a percent of total lane signal. b. COs. Left: blots of XhoI digests probed with ARG4 sequences. Right: CO2 frequencies plotted as a percent of total lane signal. c. NCOs. Left: blots of XhoI/EcoRI digests probed with HIS4 sequences. Right: NCO frequencies plotted as a percent of total lane signal. (TIF) [file pgen.1002083.s002.tif]
