# Supplementary material for: Meiotic Recombination Intermediates Are Resolved with Minimal Crossover Formation during Return-to-Growth, an Analogue of the Mitotic Cell Cycle
Source: PLoS Genet. 2011 May 26;7(5):e1002083. doi: 10.1371/journal.pgen.1002083 (PMC3102748; doi:10.1371/journal.pgen.1002083)
Supplement: Table S1 — Strains used in this work. All are MAT a/MATα lys2/lys2 ho::LYS2/ho::LYS2. The ndt80 allele is ndt80Δ(Eco47III-BseRI)::KanMX6. MJL2984-derived strains contain the recombination reporter illustrated in Figure 5. (DOC) [file pgen.1002083.s003.doc]

| Strain | Genotype |
| --- | --- |
| MJL2984 | *his4::URA3-tel-arg4-EcPal+9 leu2-R arg4*∆*(eco47III-hpaI) ura3*∆*(hind3-sma1)*  -------------------------------------------------- -------------------- -----------------  *HIS4 leu2-R::URA3-tel-ARG4 arg4*∆*(eco47III-hpaI) ura3*∆*(hind3-sma1)* |
| MJL3164 | *ura3* *ndt80∆::KanMX6*  MJL2984+ ---- --------------  *ura3* *ndt80∆::KanMX6* |
| MJL3267 | *ura3* *ndt80∆::KanMX6 cyh2-z* *CDC5*  MJL2984+ ---------------------------- -------------- ------ ---------------------  *ura3::Pgpd1-GAL4(848)-ER-URA3 ndt80∆::KanMX6 CYH2* *HphMX4-Pgal1-3HA-CDC5* |
| MJL3388 | *ura3* *ndt80∆::KanMX6* *sgs1*∆*::KanMX TRP1::HphMX::sgs1-*∆*C795*  MJL2984+ ---- -------------- ------------ -----------------------  *ura3* *ndt80∆::KanMX6* *sgs1*∆*::KanMX TRP1::HphMX::sgs1-*∆*C795* |
| MJL3389 | *ura3 ndt80∆::KanMX6* *mus81*∆*::KanMX*  MJL2984+ ---- -------------- --------------  *ura3* *ndt80∆::KanMX6* *mus81*∆*::KanMX* |
| MJL3428 | *ura3 spo11-Y135F-HA::URA3 ndt80*∆*::KanMX6* *sgs1*∆*::KanMX TRP1::HphMX::sgs1*-∆*C795*  MJL2984+ ---- -------------------- -------------- ------------ -----------------------  *ura3* *spo11-Y135F-HA::URA3 ndt80*∆*::KanMX6 sgs1*∆*::KanMX TRP1::HphMX::sgs1*-∆*C795* |
| MJL3430 | *ura3::Pgpd1-GAL4(848)-ER-URA3 Pgal1-NDT80:TRP1*  MJL2984+ ---------------------------- ----------------  *ura3::Pgpd1-GAL4(848)-ER-URA3 Pgal1-NDT80:TRP1* |
| MJL2807 | *ndt80*∆*::KanMX6 spo11-Y135F-HA::URA3*  *-------------- --------------------*  *ndt80*∆*::KanMX6 spo11-Y135F-HA::URA3* |
| MJL3163 | *ndt80*∆*(Eco47III-BseRI)::KanMX6* *trp1::hisG*  ------------------------------ ----------  *ndt80*∆*(Eco47III-BseRI)::KanMX6* *TRP1* |
| MJL3312 | *leu2-RV::URA3-(Sma1-Eco47III)-[ARG4] ndt80*∆*::KanMX6 CENV*  ------------------------------------ -------------- *------------------*  *leu2::hisG LEU2::Pura3-TetR-GFP ndt80*∆*::KanMX6 CENV:tetOx224-HIS3* |
| MJL3313 | *LEU2::Pura3-TetR-GFP CEN5:tetOx224-HIS3*  *-------------------- ------------------*  *LEU2::Pura3-TetR-GFP CEN5:tetOx224-HIS3* |
| MJL3548 | *cyh2-z* *TRP1*  ------ ----------  *CYH2 trp1::hisG* |

Table 1. Strains used in this work. All are *MAT***a**/*MATα* *lys2/lys2 ho::LYS2/ho::LYS2*. The *ndt80* allele is *ndt80*∆*(Eco47III-BseRI)::KanMX6*. MJL2984-derived strains contain the recombination reporter illustrated in Figure 5.
